# Assessing practice pattern differences in the treatment of acute low back pain in the United States Military Health System

**DOI:** 10.1186/s12913-018-3525-8

**Published:** 2018-09-17

**Authors:** Erich J. Dietrich, Todd Leroux, Carla F. Santiago, Melvin D. Helgeson, Patrick Richard, Tracey P. Koehlmoos

**Affiliations:** 1 Uniformed Services University of the Health Sciences, 4301 Jones Bridge Road, Bethesda, MD 21779 USA; 2 Naval Hospital Okinawa, Chatan, , Nakagami District, , Okinawa Prefecture 904-0103 Japan; 3 Walter Reed National Military Medical Center, Bethesda, 20814 USA

**Keywords:** Physician extenders, Low back pain, Military medicine, Health services, Practice guideline, Nurse practitioner, Physician assistant

## Abstract

**Background:**

Acute low back pain is one of the most common reasons for individuals to seek medical care in the United States. The US Military Health System provides medical care to approximately 9.4 million beneficiaries annually. These patients also routinely suffer from acute low back pain. Within this health system, patients can receive care and treatment from physicians, or physician extenders including physician assistants and nurse practitioners. Given the diversity of provider types and their respective training programs, it would be informative to evaluate variation in care delivery, adherence to clinical guidelines, and differences within the MHS among a complex mix of provider types.

**Methods:**

This study was a retrospective, cross-sectional quantitative analysis that examined variations in treatment between provider types within the Military Health System in 2015 for treatment of acute low back pain using administrative data. In addition to descriptive and summary statistics, binomial logistic regression models were used to assess variation in practice patterns among physicians and mid-level practitioners for prescribing of non-steroidal anti-inflammatory, opioids, plain radiography, computed tomography, and magnetic resonance imaging.

**Results:**

With regard to prescribing practices, results indicated that the odds of receiving non-steroidal anti-inflammatory prescriptions increased significantly for both physician assistants and nurse practitioners when compared to physicians. For basic radiological referrals, odds increased significantly for ordering plain radiography for physician assistants and nurse practitioners when compared to physicians. For more advanced imaging, odds significantly decreased for ordering computed tomography (CT) and slightly decreased for magnetic resonance for physician assistants, nurse practitioners and physician residents compared to the physician group. Additionally this study discovered differences in the prescribing patterns between provider categories. Both contractors and civilians had higher odds of prescribing opioids compared to active duty providers.

**Conclusions:**

As physician assistants and nurse practitioners continue to gain popularity as physician extenders in the US and in addressing provider shortages for the Military Health System, further research should be conducted to determine what impact, if any, the differences found in this study have on patient outcomes. In addition, provider type warrants further investigation to determine if labor mix and outsourcing decisions within a single payer system impacts health delivery and value based care.

## Background

Lower back pain is estimated to affect up to 80% of all adults during their lifetimes [1], and accounts for over $100 billion in yearly costs, due largely to lost work and productivity [2]. Multiple experts, such as the American College of Physicians (ACP) and the American Society of Anesthesiologists (ASA), conclude that the use of imaging with plain radiography, computed tomography (CT), or magnetic resonance imaging (MRI) does not improve patient outcomes for non-specific low back pain not otherwise attributed to an underlying condition [3]. These clinical practice guidelines provide a standard of care which is evidence-based and provides a practical approach to assessing, diagnosing, and treating patients who present with acute low back pain [4]. The Department of Defense (DoD) and Veterans Affairs (VA) medical systems [5] published similar clinical practice guidelines in 2007 (subsequently revised in 2017), using key indicators developed by the American College of Physicians (ACP) and the American Pain Society (APS).

The U.S. Military Health System (MHS) makes use of a mixed workforce including active duty providers, civilian General Schedule (GS) employees and contractors, which fosters both improved beneficiary care and overall military readiness. The system also relies heavily on mid-level practitioners, known as physician extenders, to provide medical care for its beneficiaries [6].These physician extenders include nurse practitioners (NPs) and physician assistants (PAs), who require advanced educational degrees, and are considered largely equivalent in practice. Differences between the two include that PAs follow a medical model and must practice under physician oversight, while NPs follow a nursing model, and may practice without physician oversight even in specific clinical specialties [7]. Evidence suggests that patients treated by NPs and PAs have similar outcomes as those treated by physicians [8–10]. It is assumed that care delivered by active duty, civilian, or contracted providers is comparable; however, this assumption enables naïve cost benefit comparisons based solely on salary and benefits, without taking into account the difference in the quality of care provided by providers with different training and specialization. Active duty providers generally have fewer years of practice and are closer to their training and more likely well versed in the latest clinical practice guidelines. As the MHS must maintain alignment between the dual missions of delivering beneficiary care and maintaining provider capability to address war time challenges (known as ‘readiness’), total workforce mix decisions become more complex than they appear, and such factors are frequently not considered by official MHS policy documents [11].

Therefore, the focus of this study is to examine differences in practice between provider categories across the MHS workforce, as well as between physicians and physician extenders, in providing guideline-concordant imaging and prescription practices for treatment of low back pain. The outcome of this study, using clinical practice guidelines as a benchmark, will help to inform and promote more accurate, evidence-informed personnel management, which in turn will foster greater access and more effective care for both service members and their beneficiaries.

## Methods

### Population

The MHS provides medical treatment to approximately 9.4 million US military service members, their families, and retirees [12], and has two mechanisms for healthcare delivery. Direct care is provided to beneficiaries in military hospitals and clinics. Purchased care encompasses care in the civilian sector, and is associated with TRICARE health insurance. Due to perceived differences in obtaining medical care in different countries served by the MHS, the search was limited to patients and providers in the direct care system within the United States.

### Data source

Data for this study were extracted from the Military Health System Data Repository (MDR) for adult patients (between ages 18–64) with an International Classification of Disease Ninth Edition (ICD-9) diagnosis code of nonspecific low back pain (724.2). We considered adding additional ICD-9 codes, but this would have further exposed our analysis to additional uncontrollable variables. For example, adding ICD-9 codes specific to radiculopathy could expose us to additional red flags which would not make our analysis valid for imaging. Furthermore, a vast majority of patients presenting with acute low back pain were coded for code 724.2, creating an appropriate sample size. Specifically, we identified 288,077 patients diagnosed with lumbago (ICD-9724.2) in fiscal year 2015. A six month walk-back from the initial diagnosis date was used to ensure an acute incidence and no previous history of lumbago, resulting in 244,333 acute low back pain patients. Further measures were used to remove data quality issues with the reported provider specialty, and only encounters with Physicians, Physician Assistants, Nurse Practitioners, and Physician Residents were selected for our final study populations and analyses; 195,844. Treatments (NSAIDs, Opiates, CT, Radiography, and MRI) were matched to acute low back pain encounters within four weeks of diagnosis date, consistent with the DoD/VA Clinical Practice Guideline. In FY 2015, the patient population with ICD-9724.2 were treated by 10,232 providers.

Treating provider characteristics include provider type (physician, NP, or PA), clinical specialty, military branch of service (Army, Navy, or Air Force), personnel category (active duty, civilian, contractor), and the TRICARE region where the provider is employed. Patient characteristics include age, gender, beneficiary category (active duty, retiree, or dependent), and marital status.

### Measurement indicators

The DoD/VHA Clinical Guidelines for this diagnoses and treatment were applied in this study as a benchmark for comparing provider type and category. Specifically, identifying if imaging or pharmaceuticals were prescribed within four weeks of an initial diagnosis of Lumbago. In narrowing our focus to a single ICD-9 diagnosis and only the first four weeks of treatment, we isolated a very specific part of the CPG for adherence. The imaging procedures were selected from the DoD/VHA Clinical Guidelines for diagnosis and treatment of low back pain include radiography, CT, or MRI, which were captured at the provider level, and the proportion attributable to each provider for these categories was also captured. The same process was performed to identify factors associated with prescription of NSAIDs or opioids for low back pain.

### Statistical analysis

Separate binomial logistic regression models were used to calculate the odds for prescribing non-steroidal anti-inflammatory drugs (NSAID), opiates, plain radiography, computed tomography (CT), and magnetic resonance imaging (MRI). These models were adjusted for provider military service (Army, Navy, Air Force, and Other), personnel category (whether the provider was military Active Duty, a Civil Servant, Contractor, or Other), and geographic region (in 2015, TRICARE had three regions in the Continental United States that include West, North, and South). Models were adjusted for patient characteristics, to include age, gender, and beneficiary category (Active Duty, Military Dependent, Military Retiree, and Inactive Guard / Reserve). Data management and statistical analyses were performed using SAS software.

## Results

A total of 10,232 providers treated patients for low back pain in 2015, with greatest representation among physicians (61.9%), active duty personnel (66.3%), and Army service (46.2%). Non-physician providers included PAs (20.5%), NPs (12.1%), and physician residents (5.5%) (Table 1).

**Table 1:** Demographic Characteristics of Providers

Characteristic	Providers
	FY 2014 (*n* = 10,232)
	n (%)
Provider Type	
Physician	6337 (61.9)
Physician Assistant	2095 (20.5)
Physician Resident	562 (5.5)
Nurse Practitioner	1238 (12.1)
Military Service	
Army	4723 (46.2)
Air Force	2628 (25.7)
Navy	2422 (23.7)
Other	459 (4.4)
Personnel Category	
Active Duty	6665 (65.1)
Civilian	2122 (20.7)
Contractor	949 (9.3)
Other	443 (4.3)
Missing	53 (0.6)

Significant statistical differences between extenders and physicians were found in several areas, as depicted in Table 2. For NPs compared to physicians, the odds increased by a factor of 1.21 for prescribing NSAID, and a factor of 1.15 for prescribing plain radiography. The odds decreased significantly, by a factor of .43 for ordering CTs. There were marginally significant statistical differences in ordering MRIs; odds decreased by a factor of .93 and opiate prescriptions, an odds decrease by a factor of .82. For PAs compared to physicians, the odds increased by a factor of 1.38 for prescribing NSAID; and factor of 1.25 for ordering plain radiography. Similar to NPs, there was a significant decrease, by a factor of .58 for ordering CTs and marginal decrease for prescribing opiates and ordering MRIs, .92 and .94 respectively. In contrast, physician residents were more conservative in their practices across the board, and being much less likely than physicians to order CTs NSAIDS, opioids or imaging (Table 2).

**Table 2:** Prescribing Practices by Provider Type

	NSAID Prescriptions OR (95% CI)	Opiate Prescriptions OR (95% CI)	Plain Radiography OR (95% CI)	Computed Tomography OR (95% CI)	MRI OR (95% CI)
Provider Type					
Physician	1	1	1	1	1
Nurse Practitioner	1.21 (1.18–1.24)*	0.82 (0.80–0.85)*	1.15 (1.11–1.19)*	0.43 (0.33–0.58)*	0.93 (0.87–1.00)*
Physician Assistant	1.38 (1.35–1.41)*	0.92 (0.89–0.94)*	1.25 (1.22–1.28)*	0.58 (0.48–0.71)*	0.94 (0.89–0.99)*
Physician Resident	0.81 (0.75–0.86)*	0.72 (0.66–0.79)*	0.84 (0.77–0.93)*	0.35 (0.16–0.80)*	0.77 (0.63–0.93)*
Personnel Category					
Active Duty	1	1	1	1	1
Civilian	0.98 (0.96–1.01)	1.15 (1.11–1.18)*	1.06 (1.04–1.09)*	0.57 (0.46–0.70)*	0.86 (0.82–0.91)*
Contractor	1.02 (0.99–1.05)	1.29 (1.25–1.34)*	1.09 (1.05–1.13)*	1.16 (0.91–1.48)	0.98 (0.91–1.06)
Other	0.99 (0.91–1.07)	1.09 (0.99–1.21)	1.02 (0.93–1.12)	0.25 (0.08–0.85)*	0.92 (0.76–1.11)

Adjusted Odds Ratio and 95% Confidence Intervals for FY 2015

*CI* Confidence Interval; *P P*-value * *P* < .05

All models controlled for provider characteristics (military service, personnel category, TRICARE region) and patient characteristics (age, gender, beneficiary category). In all models, physicians were the reference category

Similarly, there were statistically-significant differences for civilian providers and contractors vs. active-duty physicians. Civilians and contractors had an increased odds of prescribing opiates by a factor of 1.15 for civilian providers and 1.29 for contract providers when compared to active duty providers. There were minor differences for ordering plain radiography, an increased odds of 1.06 for civilians and 1.09 for contractors compared to active duty. In addition, contracted providers ordered CTs imaging more frequently than did active-duty providers. The odds increased by a factor of 1.16. Civilian providers had a decreased odds of ordering CTs compared to active duty, by a factor of .57.

## Discussion

This study found that in comparison to physicians, physician extenders were more likely to use plain radiography imaging services, equating to greater healthcare imaging utilization and a potentially lower value of care compared to physicians for these services. When it comes to more advanced imaging, physician extenders are less likely to order CTs, and have similar patterns to physicians for ordering MRIs. This is also the case with prescribing opiates, as all three categories, NPs, PAs, and residents were less likely to prescribe these within four weeks of initial diagnosis. The results for ordering plain radiographies are consistent with a human capital model, which suggests that physicians will comply more closely with the DoD / VHA Clinical Guidelines for treatment of non-specific low back pain than will physician extenders, because of physicians’ additional years of medical education and training [13]. However, this does not explain the physician group’s increased use of opioids.

Multiple previous studies show similar outcomes and minimal variation in treatment between physicians and physician extenders [8–10]. The physician residents had practice patterns that were similar to physician extenders and with the exception of NSAIDs and CT imaging, were close to physician patterns. Taken together, this suggests that our study findings may be generalizable to other conditions treated within the MHS and with a few exceptions, variation was low for MRIs and opioid prescriptions. This is a promising finding as the MHS strives for more value based care and standardized evidence based treatment.

This study also found that, when comparing provider categories by employment type, both government civilians and contractors were more aggressive than active duty providers in prescribing opiates and ordering plain radiography imaging. These results are also noteworthy in light of recent research showing that many long-term users of opioids originally received prescriptions for low back pain [14]. However, data is insufficient to correlate long-term usage solely with the actions of civilian or contracted providers.

While this result does not indicate inappropriate care by any provider category, it does highlight the variation across provider categories in the MHS for the treatment of back pain. This is in line with a recent review of the MHS which demonstrates variations in the use and provision of healthcare [15]. This finding may be helpful to MHS decision makers when considering the staffing mix and the size of the active duty health service workforce during a time of significant change within the Defense Health Agency and MHS. Further research is necessary, as the complex array of traditional and mid-level providers among numerous provider categories, makes understanding variations in practice patterns crucial to maximizing value and patient outcomes in the MHS.

### Limitations

One limitation of this study is the absence of more detailed provider characteristics, such as years of experience, academic institution, medical residency training, and additional education or certifications, which information would support the human capital hypothesis. Similarly, while the MHS knows exactly how many active duty and GS employees work in the system at a given time, the number of contractors is not readily visible, due in part to their being managed at the local level. Further limitations include the possibility of duplicated patient encounters due to coding errors, and the acknowledgement that results from a single set of indicators on one condition may not be generalizable across all conditions treated in the MHS. It is also difficult to know if physicians treated more complex or acute cases of initial non-specific low back pain. It is also possible that non-specific back pain was coded differently than IDC-9 code 724.2, therefore additional observations may have been lost. This study is based on retrospective observational data, which limits causal links, while significant associations were identified, the results do not imply causation between provider category and adherence to clinical practice guidelines, nor do they indicate the presence of inappropriate care by providers.

### Future directions

This study is the first of its kind to look at the following of CPGs in the Military Health System by both provider type and employment category. The results of this study reveal that variations among provider type and category exist in the MHS and warrant further in-depth analyses. Furthermore, studies on mid-level practitioners should be widened to encompass a broader patient group, including those with chronic diseases. To inform policy for appropriate and optimal utilization of health resources, future research should continue to measure quality of healthcare using existing measures, such as the Quality Indicators developed by the Agency for Healthcare Research and Quality [16], as well as to investigate other important factors such as outcomes, costs, and potential unintended consequences of workforce mix decisions.

## Conclusions

The analysis in this work challenges the automatic assumption of standardized care between active duty and civilian provider types and between physicians and extenders, and investigates differences in provision of imaging and medication for non-specific lower back pain. With a global shortage of primary care doctors and a move toward using more non-physician clinicians, the use of evidence-based standardized care processes, such as clinical practice guidelines, can improve consistency between provider groups. The results of this study support the use of clinical practice guidelines and validate the process of improving quality of care through the implementation of evidence-based care.

## Abbreviations

DoD: Department of Defense
MDR: Military Health System Data Repository
MHS: Military Health System
NP: Nurse Practitioner
NSAID: Non-steroidal anti-inflammatory drug
PA: Physician Assistant
VHA: Veterans Health Affairs
